# Efficacious, effective, and embedded interventions: Implementation research in infectious disease control

**DOI:** 10.1186/1471-2458-8-343

**Published:** 2008-10-01

**Authors:** Pascale Allotey, Daniel D Reidpath, Hashim Ghalib, Franco Pagnoni, William C Skelly

**Affiliations:** 1 Centre for Public Health Research/School of Health Sciences, Brunel University, West London, UK; 2 UNICEF/UNDP/World Bank/WHO Special Programme for Research and Training in Tropical Diseases (TDR), World Health Organization, Geneva, Switzerland; 3 Centre for Public Health Research/Institute for the Environment, Brunel University, West London, UK

## Abstract

**Background:**

Research in infectious disease control is heavily skewed towards high end technology; development of new drugs, vaccines and clinical interventions. Oft ignored, is the evidence to inform the best strategies that ensure the embedding of interventions into health systems and amongst populations. In this paper we undertake an analysis of the challenge in the development of research for the sustainable implementation of disease control interventions.

**Results:**

We highlight the fundamental differences between the research paradigms associated with the development of technologies and interventions for disease control on the one hand and the research paradigms required for enhancing the sustainable uptake of those very same interventions within the communities on the other. We provide a definition for implementation research in an attempt to underscore its critical role and explore the multidisciplinary science needed to address the challenges in disease control.

**Conclusion:**

The greatest value for money in health research lies in the sustainable and effective implementation of already proven, efficacious solutions. The development of implementation research that can help provide some solutions on how this can be achieved is sorely needed.

## Background

Communicable diseases in general and parasitic and infectious diseases in particular thrive because they exploit human behaviour, and the nature of the environment, societies and culture. In the poorest regions of the world, persistent poverty, poor living conditions and environmental factors enhance the success of these diseases which continue to be a major health burden. Moreover, social, economic and political inequalities interact in complex ways to affect vulnerability, access to treatment, and the sequelae of disease. Poverty and its associated social, cultural and environmental contexts are also the critical, overarching factors that influence the impact and instrumental value of interventions including their acceptability, accessibility, affordability and sustainability.

**Figure 1:**
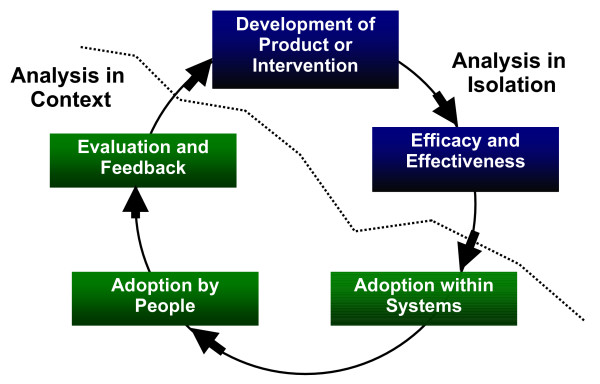
Product Development Cycle.

Research is a significant part of the strategy for the control of communicable diseases with the largest tranche of funding going to the development of new drugs, vaccines and clinical interventions. Success, particularly in drug and vaccine development, is unfortunately slow and failure is expensive [1]. Nonetheless, high-end technology continues to capture the imagination of funding bodies, in part because it is based on methodologies that are 'tried and true', including established research tools to demonstrate efficacy and the econometric techniques of cost effectiveness. It is also an area of investment that holds potential for direct economic return based on the sale of the developments [2]. However, at least as critical in the product development cycle (see figure 1) is the step, often ignored in public health, that lies between the ascertainment of effectiveness and the final sustained adoption of an intervention [3-5]. This is underscored by the fact that interventions currently exist for many high burden diseases but the conditions persist because of a failure in the uptake of the interventions by the communities that most need them. Indeed we would argue that even a single iteration of the product development cycle is not complete until (i) it has been scaled up to the end users and (ii) there is a further opportunity to contribute to ongoing product developments.

In this paper, we present an analysis of the challenges posed by those less well developed segments of the development cycle; the research for sustainable implementation of disease control interventions. We highlight the fundamental differences between the research paradigms associated with the development of technologies and interventions for disease control on the one hand, and the research paradigms required for enhancing the sustainable uptake of those very same interventions on the other. We provide a definition for implementation research in an attempt to emphasize its critical role and highlight the multidisciplinary science needed to address the challenges in disease control. We conclude by noting some key areas for attention.

### Product development, efficacy and effectiveness: Analyses in isolation

Science has traditionally been seen to advance in virtue of our ability to make inferences about causal relationships once extraneous, associated factors have been taken into account. The strongest, most persuasive evidence of a causal relationship comes from experiments in which most factors are held constant, and only those few factors under investigation for their causal properties are isolated and systematically manipulated. At one end of the health technologies development spectrum, researchers have almost complete control over the experimental environment by creating the "ideal conditions" in which to establish a cause-effect relationship. As the drug or product progresses into human trials, researchers forsake more and more physical control of the experimental environment for statistical control. The intention however, remains constant; to be in a position to analyze the causal effect of the factors of interest (the intervention) in isolation from the effects of any extraneous factors. This principle of analysis in isolation underlies the methodology for clinical trials to determine the efficacy of treatments, cures and preventive measures for disease control.

Once the *efficacy *of an intervention is established, the focus of research shifts to *effectiveness*: the causal relationship between the intervention and the outcome in humans in routine clinical care. The principle of analysis in isolation remains, although the researchers' capacity to actually ensure it decreases.

Unfortunately, knowing that a treatment is effective in routine clinical care is still insufficient, particularly in resource poor settings. The goal for rolling out treatments for infectious and parasitic disease control programs must be the sustainable adoption of the intervention by the health systems and the target population, and not simply the establishment of effectiveness in a monitored clinical population. In other words, an intervention must become *embedded; *firmly integrated as part of the health system and the health culture of the disease endemic setting. It must be available, acceptable, accessible and affordable to those who need it; used appropriately, and become a part of the disease prevention, treatment seeking culture. Where relevant, the intervention program must become institutionalised regardless of organisational or political change [6]. Interventions become embedded not by understanding cause-effect relationships in isolation from extraneous factors. Interventions become embedded through the manipulation of these contextual factors that enhance the sustainability, uptake and performance of the intervention. The noise – that is the factors that need to be 'controlled' in efficacy and effectiveness research – thus becomes the signal to be understood in implementation research.

### Embedding interventions: Analyses in context

Understanding the complexity of factors that affect whether efficacious and effective interventions become embedded is a necessary and a critical part of a complete product development cycle. However, this phase of the cycle, which falls under the rubric of 'implementation research', is deplorably underdeveloped in public health. A major review of implementation research within the context of public policy found that it is considered a well researched and relevant area in disciplines such as politics, public policy and public administration [7]. However publication is so diffuse across disciplines (including health related disciplines) that there is still no coherent body of knowledge or theoretical framework to define it [7]. This is partly due to the lack of a clear definition and operationalisation of implementation research. We define implementation research in this context as:

applied research that aims to develop the critical evidence base that informs the effective, sustained and embedded adoption of interventions by health systems and communities. It deals with the knowledge gap between efficacy, effectiveness and current practice to produce the greatest gains in disease control.

Implementation research involves the systematic and critical investigation and analysis of the dynamic, contextual processes that influence how individuals, populations and health systems change in order to adopt new technologies and interventions. The focus begins conceptually with an intervention which (from efficacy and effectiveness trials) is expected to deliver health gains, and systematically describes and analyses the process and outcomes from pre-intervention to successful adoption or indeed failure of the program, through the development and testing of approaches that support the scale up of disease control programs.

Historically, underutilisation of implementation research in public health arose because (a) the development cycle for parasitic and infection disease interventions typically only included efficacy and effectiveness; (b) implementation research does not have the status of a 'serious' science (marking a dispute over disciplinary boundaries and territory) [7]; (c) as a consequence of (a) and (b) when implementation research is deployed, it is on an ad hoc basis and not as part of a coherent strategy for disease control; and (d) as a result of the complex factors that define the reality of embedding interventions, the outcomes can often not be as clearly specified as outcomes resulting from analyses in isolation [6]. Furthermore, implementation research is seen as expensive and therefore does not attract funding and findings are often seen as contextually specific and therefore not generalisable.

### Challenges in the development of implementation research for infectious disease control

There is a growing recognition of the need to move from research to practice in the area of health technologies and clinical guidelines [8,9]. Translational research is a concept similar to implementation research and is used more commonly in the health literature. It is used in the context of translating basic laboratory based sciences to clinical application (bench to bedside) and in clinical practice guidelines [10]. It has also been used to describe tools for monitoring the process from efficacy to effectiveness trials [11-14]. In the context of resource poor settings, it has been used to describe the scale up of the delivery of treatment to HIV positive patients [15]. Sanders and Haines use the term implementation research and describe it as a subset of health systems research, locating it within the broader framework of evaluation research and focusing on how to promote the uptake and successful execution of evidence-based interventions and policies that have been identified through systematic reviews [5]. Similarly, in the editorial of the inaugural issue of Implementation Science, Eccles describes implementation research as scientific study of methods to promote the systematic uptake of research findings and other evidence-based practices into routine practice, and, hence, to improve the quality and effectiveness of health services and care [16]. While these approaches highlight progress in enhancing the significance of implementation as part of the product development cycle, there remains an almost exclusive focus on effectiveness and therefore on the delivery of personal health services. This focus continues to exclude the complexities of the broader context in dealing with population health and interventions that address access, equity, community engagement, empowerment and participation. They also do not take account of the broader issues of global and national contexts and the influences on policy, logistics of supply, local economies and broader health systems all of which are critical components to ensure embedding. Product developers need to take this seriously, because an effective intervention that cannot become embedded is a waste of resources which are invariably limited.

We would argue that implementation research for parasitic and infectious disease control, involves much more than a form of social or health program evaluation [5,17]. The problem is illustrated with tuberculosis control. The directly observed therapy, short-course (DOTS) strategy has been the key intervention for tuberculosis control promoted by the WHO since 1991. It consists of a package of separate interventions: detection of smear positive pulmonary tuberculosis, directly observed chemothearapy, guaranteed drug supply and a tracking of compliance and outcomes.

It is a vertical program with a single disease focus which should make it amenable to analysis in isolation. However, like many public health interventions, it is also a complex intervention because it relies on more than one "technology" each with a different set of extraneous variables and each introduced at different points in time by different sectors of the health system. It involves the willingness and ability of governments to adopt the policy recommendations, which in turn depend on, amongst other things, a range of political and economic factors. It involves the existence of a functioning health system which means human and physical infrastructure, a guaranteed supply chain of diagnosistics and treatment, a workforce able to undertake surveillance and service provision based on recommended guidelines, a regulation of the private sector should they be involved in the national health system and so on. There are also issues of access for the target population that include physical, geographic, social, cultural, economic factors that may enhance or impede their ability to actively seek diagnosis and treatment. Evaluation of the outcomes based on the standard measures is therefore difficult and despite being one of the longest running interventions in global health, there is no definitive data on the effectiveness of DOTS as an intervention [18]. More specifically, in the absence of the implementation research data and an analysis of the process [19], tuberculosis remains a major burden and further advances in the control effort are constrained.

Guldbransoon (2008) offers a framework for exploring the implementation process. The key concepts suggested are the intervention or product; the decision to adopt the product (for our purposes the policy development process); the planning toward the change and integration which also requires an analysis of resources; the change, signifying an increase in the level of knowledge, and a change in organisational capacity and finally an integration and institutionalisation of the intervention [p14]. Other models and frameworks exist [20] but these have been developed largely on the basis of single interventions. The area clearly needs major inputs to determine how to apply lessons across contexts [21].

Implementation research provides fertile ground for the development of interdisciplinary science across several disciplines of applied social sciences in public health. Borrowing from these multidisciplinary approaches, implementation research involves multiple methods: multilevel case studies on the feasibility of implementation, cultural and social relations in the community; appropriate targeting of limited resources, practice and policy factors, external influences, other health, social and development priorities, and monitoring and evaluation of processes and economic drivers. It also involves the analysis of intra-governmental issues around power and responsibility, as well as the relationships between government and non-government agencies and donors. These describe what should be a seamless link between research and control programmes.

The need for interdisciplinary approaches in implementation research is illustrated in a number of the successes and failures of tropical disease control programs. The efficacy and effectiveness of insecticide impregnated bed nets and curtains are a case in point. Phase III clinical trials in the early 1990s in "community health laboratories" demonstrated success in reducing mortality from malaria and morbidity and from a number of other vector borne conditions [22-24]. Ongoing monitoring also showed that the 17% reduction in mortality persisted in controlled communities seven years after the introduction of the intervention [25]. However, embedding insecticide impregnated bed nets and curtains outside these clinical trials sites continues to meet with only limited success [26].

Anthropological research has highlighted the social, economic and cultural constraints on bed net usage that is so important to sustainable uptake in the broader community [27]. From the disciplinary perspective of health economics research, the critical need to understand and account for contextual factors in bed-net uptake is highlighted through studies in health financing, cost effectiveness [28] and willingness-to-pay [29].

Health psychology and health promotion approaches have been used to influence factors that enhance the acceptability of interventions through social marketing [30]. Involving communities in decision making, again through the use of a range of social research methods, has been important in the implementation of home management of malaria in children. In a project that involved mothers in the design of medication packs appropriate to the literacy level of women in the community, for instance, Chinibua et al found that they were able to overcome the ongoing problem of compliance [31]. Working with communities has also been a key factor in the successful fight against river blindness.

## Discussion

The need for implementation research has been recognized [5,32-34] although the explicit support in global health has been slow and there has been limited engagement with product developers. Limited ad hoc funding is often provided for social science components of research and disease control programs, but these are inevitably as adjunct research to the "more serious" effectiveness research. While this may be useful for specific cases, the ultimate goal of implementation research needs to be the development of an evidence base which would allow some generalization beyond individual communities and enable predictions to be made about how well interventions would be embedded given particular conditions. The science of implementation research needs to be developed as an integrated package that promotes engagement between researchers in product development and the social scientists, disease control personnel and communities that will benefit from the interventions.

Development of capacity in this area has to be a critical component of the renewed commitment to tackle neglected parasitic and infectious diseases. The Special Programme for Research and Training in Tropical Diseases (TDR) has supported the development of training at different levels of capacity [35] in Ghana and Kenya to enable researchers from endemic countries to develop not only the theoretical and research skills required in implementation research, but also the leadership abilities to address an area that is inherently political and challenging (not least because of demarcation and guild motivated disputes between disciplines). These programs are further supported by short courses to train field researchers and the potential future workforce in implementation research.

## Conclusion

The benchmark statement of the Declaration of Alma Ata provides a cogent argument for evidence based solutions that are practical, socially acceptable, accessible, and available at a cost that is sustainable. A commitment to alleviating the burden of parasitic and infectious diseases in resource poor settings is incomplete if it is does not close the product development cycle, from the basic sciences to the embedding of interventions. The greatest value for money in health research lies in the sustainable and effective implementation of already proven, efficacious solutions and less in the development of new tools and products for which the implementation research remains to be done. The development of the research that can help provide some solutions on how this can be achieved is sorely needed [21].

## Competing interests

The authors declare that they have no competing interests.

## Authors' contributions

PA led on the conceptualisation and writing of this paper. DDR, HG, FP, WCS, all contributed to the writing and editing of this paper.

## Pre-publication history

The pre-publication history for this paper can be accessed here:


